# Large Thrombus Entrapped in a Patent Foramen Ovale during Inferior Vena Cava Filter Protection for Venous Thromboembolism

**DOI:** 10.1155/2023/8829652

**Published:** 2023-08-31

**Authors:** Eiji Nakamura, Kazuyoshi Takagi, Kousuke Saku, Shinya Negoto, Tomoyuki Anegawa, Shinichi Imai, Hiroyuki Otsuka, Shinichi Hiromatsu, Eiki Tayama

**Affiliations:** Division of Cardiovascular Surgery, Department of Surgery, Kurume University School of Medicine, Kurume, Japan

## Abstract

*Background*. A large thrombus entrapped in the patent foramen ovale (PFO) is an extremely rare condition. Moreover, it is considered even rarer after temporary inferior vena cava filter (TIVCF) placement for the prevention of fatal pulmonary embolism due to venous thromboembolism (VTE). *Case Report*. A 58-year-old man presented with syncope following chest pain and dyspnea due to PE exacerbation during TIVCF protection, which then led to cardiogenic shock. Echocardiography revealed a large thrombus entrapped in the PFO, and computed tomography (CT) showed a bilateral pulmonary artery embolism. The patient was treated with open surgical embolectomy for a pulmonary artery thrombus and PFO thrombus with simultaneous closure of the PFO. The patient's postoperative course was uneventful. *Results and Conclusion*. Surgical embolectomy was useful with respect to the feasibility of resection of both intracardiac thrombus and pulmonary artery thrombus performed simultaneously, contributing to the prevention of systemic embolisms, and echocardiography plays an important role for early diagnosis.

## 1. Introduction

Pulmonary embolism (PE) with intracardiac thrombus entrapped in a patent foramen ovale (PFO) is an extremely rare and critical condition because of the increasing incidence of paradoxical embolism and sudden death [1–5]. A temporary inferior vena cava filter (TIVCF) is placed to prevent PE from deep vein thrombus (DVT) of the lower extremity, and no case of recurrent PE during TICVF protection was reported [6]. Herein, we present a very rare case of a large thrombus entrapped in a PFO with second attack PE during the placement of TICVF for venous thromboembolism (VTE).

## 2. Case Report

**Figure 1:**
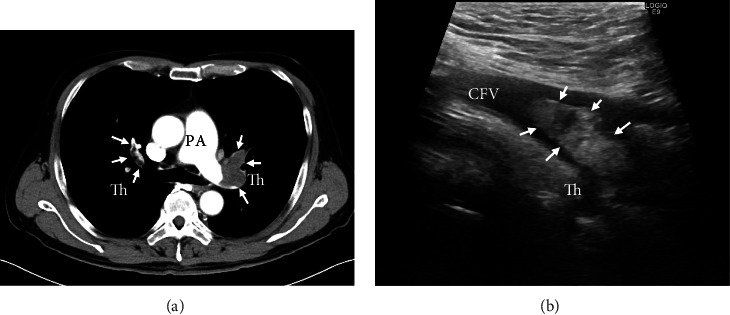
Previous CT and venous echography findings. Previous CT showed bilateral PE (white arrows (a)) and venous echography showed a mobile-shaped thrombus (white arrows) at the proximal site of the left common femoral vein (b). CT: computed tomography, PE: pulmonary embolism, PA: pulmonary artery, CFV: common femoral vein, Th: thrombus.

A 58-year-old man was admitted to another hospital with a 1-month history of dyspnea and shortness of breath without any specific cause. Computed tomography (CT) showed PE in the bilateral pulmonary artery (PA) and venous thrombus from the left popliteal vein to the left common femoral vein (CFV) (Figure 1(a) and 1(b)). Venous echography also revealed a floating venous thrombus at the proximal site of the CFV. Transthoracic echocardiography (TTE) showed that the left ventricle was compressed due to the dilatated right ventricle with pulmonary hypertension (estimated systolic pulmonary artery pressure (sPAP): 65 mmHg). PFO and intracardiac thrombi could not be detected in TTE at that time. An ALN inferior vena cava (IVC) filter (ALN, Bormes les Mimosas, France) was placed immediately into the infrarenal IVC to prevent extensive PE due to a residual floating venous thrombus at the left CFV. Anticoagulation therapy with 30 mg of rivaroxaban (Bayer, Co., Ltd, Japan) daily was then administrated. The D-dimer was 4.7 *μ*g/mL, and a predisposition to thrombotic disease was not detected.

He was then transported to our hospital one day after admission for further intensive care. Fortunately, his circulatory and respiratory states were stable with an oxygen 10 L mask and anticoagulation therapy using continuous intravenous heparin infusion with control at 1.5-2 times the normal range of activated partial thromboplastin time (APTT). He stood up first time 5 days after admission. Then, he suddenly complained of chest pain and dyspnea. He lost consciousness with arterial pressure of 60/32 mmHg, pulse rate of 95 beats per minute, and respiratory rate of 30 breaths per minute. Arterial blood gas analysis revealed hypoxia with 54.2 mmHg of partial pressure of oxygen and hypocapnia with 31.2 mmHg of partial pressure of carbon dioxide. Laboratory results showed a D-dimer level of 135.6 *μ*g/mL.

**Figure 2:**
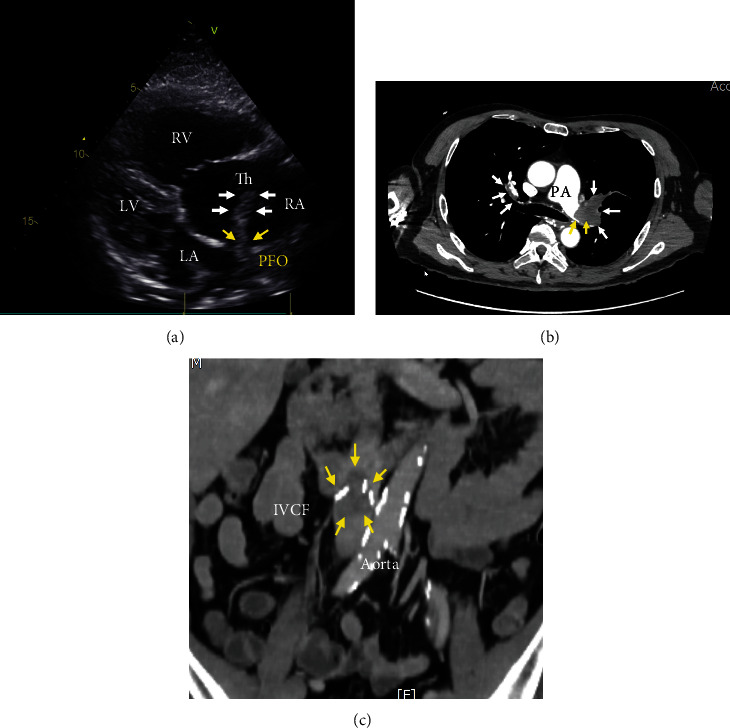
Preoperative TTE and CT findings. TTE showed dilatation of the right heart system and a newly mobile thrombus (white arrows) in the right atrium with protrusion into the left atrium through the PFO (yellow arrows (a)). CT showed extended thrombosis formation of the left main PA (yellow arrows) in addition to preexisting bilateral PE (white arrows (b)) and occluded IVCF by trapped venous thrombus (c). Yellow arrows indicated the trapped thrombus by IVCF. The thrombus was located at the proximal site of the IVCF. TTE: transthoracic echocardiography, CT: computed tomography, PA: pulmonary artery, PE: pulmonary embolism, IVCF: inferior vena cava filter, RA: right atrium, RV: right ventricle, LA: left atrium, LV: left ventricle, PFO: patent foramen ovale, Th: thrombus.

TTE showed a large thrombus, almost 5 cm in size, which was entrapped in the PFO and protruded into the left atrium (Figure 2(a)). Significant pulmonary hypertension (estimated sPAP of 80 mmHg) was also confirmed. CT showed bilateral massive PE with extensive left main PA thrombus (Figure 2(b)) and occlusion of the IVC due to a trapped venous thrombus just above the TIVCF (Figure 2(c)). The partial left superior vena cava (SVC) to the coronary sinus was accidentally confirmed. These results suggest that an isolated thrombus from the proximal site of the TIVCF flowed into the right heart system and led to cardiogenic shock due to the second attack PE.

Extracorporeal membrane oxygen (ECMO) was immediately established between the right jugular vein (JV) to the SVC and the right common femoral artery (CFA) because the infrarenal IVC was obstructed, and the partial left SVC (PLSVC) existed. Thrombolysis with 800,000 IU of monteplase (Eisai, Co., Ltd, Japan) was performed. However, a large thrombus still existed in the PFO 5 hours after thrombolytic therapy. We decided to perform an emergent surgical embolectomy.

**Figure 3:**
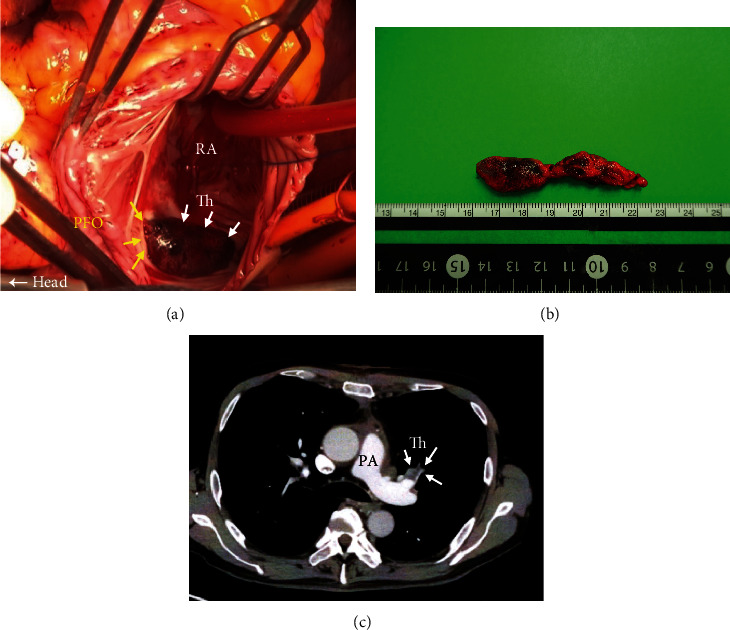
Intraoperative picture, resected thrombus, and postoperative CT findings. Intraoperative picture showed an entrapped large thrombus in the PFO (a) and resected large thrombus (b). Yellow arrows indicated the PFO, and white arrows indicated an entrapped thrombus, respectively (a). Postoperative CT showed the disappearance of the thrombus in the left main PA (c). White arrows indicated the residual thrombus in the left PA. PFO: patent foramen ovale, CT: computed tomography, PA: pulmonary artery, Th: thrombus.

Because of the bleeding risk for circulatory arrest under deep hypothermia in the preoperative shock state and after thrombolysis, and because of the relatively acute pulmonary artery thromboembolism, which was expected to improve hemodynamically once most of the proximal thrombus was removed, it was decided not to use circulatory arrest under the deep hypothermia. Cardiopulmonary bypass (CPB) with mild hypothermia was initiated using a previously placed cannula of the right CFA and JV for ECMO. An additional venous cannula to the IVC was inserted from the right atrium (RA) after median sternotomy. We performed an aortic clamp to avoid thromboembolism due to a large thrombus in the left atrium. A large thrombus entrapped in the PFO was revealed after opening the RA (Figure 3(a)). The top of the PFO was incised 1 cm upward to remove a large thrombus in an en bloc form, and then, the PFO was directly closed. The most explanted thrombi were composed of old thrombus (Figure 3(b)). Thrombectomy of the left main PA was also performed. Occluded infrarenal IVC and PLSVC did not affect the establishment of CPB and obtaining the good surgical field.

Postoperative ECMO and nitric oxide inhalation (iNO) were necessary for weaning the CPB. ECMO and iNO could be withdrawn 2 days after surgery. Continuous intravenous heparin infusion as postoperative anticoagulation therapy was started the day after surgery with control at 1.5-2 times the normal range of APTT, and 60 mg of edoxaban (Daiichi Sankyo, Co., Ltd, Japan) daily was administrated 8 days after surgery. Postoperative CT showed the disappearance of the left main PA thrombus with residual chronic thrombus at the bilateral distal PA without systemic embolism (Figure 3(c)). The estimated sPAP decreased to 28 mmHg within the normal range at discharge. He was discharged 30 days after surgery. There were no major cardiovascular events during 3 months of his postoperative follow-up.

## 3. Discussion

This is the first report of a large thrombus entrapped in the PFO with 2nd attack PE during TICVF protection.

Although a large thrombus entrapped in the PFO is an extremely rare condition [1–5], early diagnosis and treatment are very important because the mortality rate for cases with paradoxical embolism is 18%, and 66% of deaths occurring within 24 h [3]. In this case, the presence of PFO could not be detected at the first medical examination. We speculate that increased pressure in the right heart system due to PE may lead to the dilatation of PFO and the passage of thrombus into the left atrium at the second attack [4, 5]; therefore, echocardiography screening should be performed routinely for VTE, especially in patients with floating residual DVT [3].

The guidelines for diagnosis, treatment, and prevention of pulmonary thromboembolism and deep vein thrombosis (Japan Circulation Society 2017) state [7] that TIVCF is generally indicated for patients with contraindications to anticoagulation, but TIVCF may be considered for potentially fatal PE due to secondary embolization of residual thrombus, even if anticoagulation therapy is possible. Previous studies have demonstrated that the frequency of recurrent fatal pulmonary embolism during TIVCF placement is extremely rare [6, 8]. Although we established an appropriate anticoagulation therapy and placed TICVF, a large thrombus was isolated and entrapped in the PFO with a second attack PE during TIVCF placement in this case. We suppose that the secondary pulmonary embolism was the result of new thrombus formation from the nucleus of a thrombus trapped in a previously inserted IVC filter [9]. This result suggests that the possibility of a second attack PE always should be cared for in patients with DVT, especially first ambulation after PE, despite initiating the anticoagulation therapy and TIVCF protection. In addition, paradoxical systemic thromboembolism could occur during the placement of TIVCF. Even in other types of IVC filters, there is always a gap between filter struts. Thus, even if other types of IVC filters had been used, thrombotic events passing through the gap between the filter struts would not have been completely avoided.

Anticoagulation therapy, thrombolysis, and surgery are available therapeutic options; however, the best management remains controversial. Fauveau et al. reported 88 cases of entrapped thrombus in PFO in a literature review and self-examination and found that 44% of patients with paradoxical embolism and the most frequent site of systemic embolism was cerebral [5]. Geltes et al. also reported a case of thrombus in transit across a PFO complicating DVT and PE, which was discovered after a prior cerebral embolism, and was successfully treated by surgical embolectomy with closure of the PFO [10]. Therefore, they concluded that surgical treatment appears justified in the prevention of paradoxical embolism and must be performed without delay if it is the preferred treatment strategy. In fact, the entrapped thrombus in PFO was composed mainly of old thrombus, and thrombolytic therapy was not effective in this case. We believe that surgical treatment should be considered as a first-line therapy for these cases.

In conclusion, the possible risk of second attack PE and paradoxical systemic thromboembolism always should be considered during the placement of a temporary inferior vena cava filter in patients with VTE.

## Data Availability

The data used to support the findings of this study are available from the corresponding author upon request.

## Abbreviations

APTT: Activated partial thromboplastin time
CFA: Common femoral artery
CPB: Cardiopulmonary bypass
CVF: Common femoral vein
ECMO: Extracorporeal membrane oxygen
iNO: Nitric oxide
IVC: Inferior vena cava
JV: Jugular vein
PE: Pulmonary embolism
PFO: Patent foramen ovale
PLSVC: Partial left superior vena cava
RA: Right atrium
sPAP: Systolic pulmonary artery pressure
SVC: Superior vena cava
TIVCF: Temporary inferior vena cava filter
TTE: Transthoracic echocardiography
VTE: Venous thromboembolism.
